# Extracellular Vesicle Inhibitors Enhance Cholix-Induced Cell Death via Regulation of the JNK-Dependent Pathway

**DOI:** 10.3390/toxins16090380

**Published:** 2024-08-29

**Authors:** Kazuya Ozaki, Hiyo Nagahara, Asaka Kawamura, Takashi Ohgita, Sachika Higashi, Kohei Ogura, Hiroyasu Tsutsuki, Sunao Iyoda, Atsushi Yokotani, Toshiyuki Yamaji, Joel Moss, Kinnosuke Yahiro

**Affiliations:** 1 Laboratory of Microbiology and Infection Control, Division of Biological Sciences, Kyoto Pharmaceutical University, Kyoto 607-8414, Japan; ky19074@ms.kyoto-phu.ac.jp (K.O.); ky20252@ms.kyoto-phu.ac.jp (H.N.); ky20120@ms.kyoto-phu.ac.jp (A.K.); ky19278@ms.kyoto-phu.ac.jp (S.H.); ay.yokotani@gmail.com (A.Y.); 2 Center for Instrumental Analysis, Kyoto Pharmaceutical University, Kyoto 607-8414, Japan; ohgita@mb.kyoto-phu.ac.jp; 3 Division of Food Science and Biotechnology, Graduate School of Agriculture, Kyoto University, Kyoto 606-8501, Japan; ogura.kohei.7x@kyoto-u.ac.jp; 4 Department of Microbiology, Graduate School of Medical Sciences, Kumamoto University, Kumamoto 860-8556, Japan; tsutsuki@kumamoto-u.ac.jp; 5 Department of Bacteriology I, National Institute of Infectious Diseases, Tokyo 162-8640, Japan; siyoda@niid.go.jp; 6 Kyoto Biken Laboratories, Inc., Kyoto 611-0041, Japan; 7 Department of Biochemistry and Cell Biology, National Institute of Infectious Diseases, Tokyo 162-8640, Japan; t.yamaji.yx@juntendo.ac.jp; 8 Department of Microbiology and Immunology, Faculty of Pharmacy, Juntendo University, Chiba 279-0013, Japan; 9 Clinical Care Medicine and Pulmonary Branch, National Heart, Lung, and Blood Institute, National Institutes of Health, Bethesda, MD 20824-0105, USA; mossj@nhlbi.nih.gov

**Keywords:** Cholix, cell death, extracellular vesicles pathway inhibitor, RNA-seq, JNK

## Abstract

*Vibrio cholerae* is an important foodborne pathogen. Cholix cytotoxin (Cholix), produced by *V. cholerae*, is a novel eukaryotic elongation factor 2 (eEF2) adenosine diphosphate ribosyltransferase that causes host cell death by inhibiting protein synthesis. However, the role of Cholix in the infectious diseases caused by *V. cholerae* remains unclear. Some bacterial cytotoxins are carried by host extracellular vesicles (EVs) and transferred to other cells. In this study, we investigated the effects of EV inhibitors and EV-regulating proteins on Cholix-induced hepatocyte death. We observed that Cholix-induced cell death was significantly enhanced in the presence of EV inhibitors (e.g., dimethyl amiloride, and desipramine) and Rab27a-knockdown cells, but it did not involve a sphingomyelin-dependent pathway. RNA sequencing analysis revealed that desipramine, imipramine, and EV inhibitors promoted the Cholix-activated c-Jun NH2-terminal kinase (JNK) pathway. Furthermore, JNK inhibition decreased desipramine-enhanced Cholix-induced poly (ADP-ribose) polymerase (PARP) cleavage. In addition, suppression of Apaf-1 by small interfering RNA further enhanced Cholix-induced PARP cleavage by desipramine. We identified a novel function of desipramine in which the stimulated JNK pathway promoted a mitochondria-independent cell death pathway by Cholix.

## 1. Introduction

Cholix toxin (Cholix), produced by the intestinal pathogen *Vibrio cholerae*, is a mono-adenosine diphosphate ribosyltransferase cytotoxin [1]. Cholix inhibits protein synthesis in host cells via the adenosine diphosphate ribosylation of eukaryotic elongation factor 2 (eEF2) [2]. The three-dimensional structure of Cholix is highly similar to that of *Pseudomonas* exotoxin [2] and *Aeromonas* exotoxin [3], although the primary sequence of Cholix exhibits only 35–45% identity with these toxins. The Cholix-encoding gene *chxA* is found mainly in *V. cholerae* non-O1/non-O139 strains from clinical and environmental sources but not in toxigenic *V. cholerae* O1/O139 strains [4,5]. Furthermore, previous studies have detected *chxA* in *V. cholerae* non-O1/non-O139 and O1/O139 strains [6]. Awasthi et al. demonstrated that *chxA* has a prototype (*chxA* I) and variants, *chxA* II and III [4], and that ChxA I and ChxA II, but not ChxA III, are cytotoxic to various cells.

Cholix binds to low-density lipoprotein (LDL) receptor-related protein 1 and/or unknown receptors on the cell surface and is translocated to the cytosol via endocytosis [2,7]. Cleavage of a consensus sequence (RXK/RR) by Furin results in the activation of the toxin in the host cell [8]. Cholix-induced cell death is suppressed by Furin inhibitors [7]. Endocytosed Cholix migrates from the apical side to the basolateral side via vesicular transcytosis, and domain I of Cholix plays a vital role in trafficking polarized intestinal epithelial cells [9]. Liu et al. reported that the transcytosis of Cholix is regulated by several host proteins, including G protein-coupled receptor 75, transmembrane protein 132A, and Furin, which promote vesicular transcytosis across polarized intestinal epithelial cells after apical endocytosis [10].

We previously reported various mechanisms underlying Cholix-induced cell death. HeLa cells and human immortalized hepatocytes are regulated by mitochondria-dependent or -independent caspase activation, followed by poly(ADP-ribose) polymerase (PARP) cleavage (cPARP) during apoptosis, resulting in cell death [11,12]. In HepG2 cells, co-incubation of Cholix with tumor necrosis factor a (TNF-α) promotes a higher level of caspase activation compared with that using Cholix alone or TNF-α alone [13]. Prohibitin participates in mitochondrial dysfunction and cytoskeletal rearrangement during Cholix-induced cell death through direct interaction with Cholix [12]. eEF2-ribosylating toxins, including *Pseudomonas* exotoxin, Cholix, and diphtheria toxin, trigger ribosome stress and the subsequent activation of the Nod-like receptor protein 1 inflammasome [14,15].

According to the Minimal Information for Studies of Extracellular Vesicles (MISEV) guidelines, extracellular particles (EPs) are structures derived from the plasma membrane and are categorized into several groups, including extracellular vesicles, synthetic vesicles, artificial cell-derived vesicles, and non-vesicular extracellular particles [16]. Tetraspanin membrane proteins (e.g., CD9, CD63, CD81) are used as markers of extracellular vesicles (EVs) [17]. These proteins play important roles in various physiological events [18]. The biogenesis and trafficking of EVs involve several key proteins (e.g., Rab27a [19], Alix [20], SMPD2 and 3 [21,22], HGS [23]). EV secretion by host cells appears to be involved in the immune response to bacterial toxins [24,25]. Nanometer-sized cell-derived particles contain cell-type-specific proteins, lipids, and nucleic acids [26,27,28]. EVs play important roles in cell–cell communication, inflammation, and cancer diagnosis [28,29]. Shiga toxin-2 of enterohemorrhagic *Escherichia coli* or listeriolysin O of *Listeria monocytogenes* is endocytosed into target cells, and part of the cytotoxin is released from cells in exosomes [30,31].

The EV inhibitors desipramine and imipramine are used to treat psychiatric disorders [32] and also inhibit acid sphingomyelinase (SMPD1) [33]. Dimethyl amiloride (DMA) is used to treat high blood pressure and inhibits EVs by downregulating calcium channels [34]. To investigate whether EV-related pathways are involved in Cholix-induced cell death, we examined the effects of several EV inhibitors and the knockdown of genes encoding EV-regulated proteins. Additionally, we analyzed the effects of desipramine on gene expression using RNA sequencing (RNA-seq). We found that the inhibition of EV secretion or Rab27a knockdown enhanced Cholix-induced mitochondria-independent apoptosis by stimulating a JNK-dependent pathway.

## 2. Results

### 2.1. Effect of Extracellular Vesicle Inhibitors on the Cholix-Induced Cell Death Signaling Pathway

**Figure 1:**
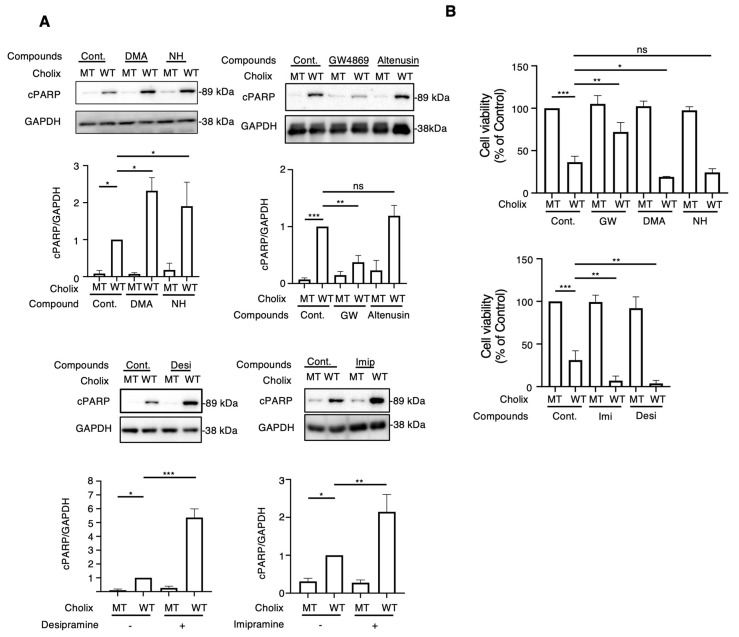
Effects of extracellular vesicle inhibitors on the Cholix-induced cell death pathway. (**A**) Hepatocytes (3 × 10^4^ cells/48-well plate) were incubated for 8 h with catalytically inactivated mutant (MT) or wild-type (WT) Cholix in the presence or absence of dimethyl sulfoxide (DMSO, control; 0.1%), 10 μM dimethyl amiloride (DMA), 10 μM neticonazole hydrochloride (NH), 10 μM GW4869, 10 μM altenusin, 10 μM desipramine (Desi), or 10 μM imipramine (Imip). Proteins from the cells were analyzed using Western blotting with an anti-cleaved PARP (cPARP) antibody. GAPDH was used as an internal control. Densitometric analysis of cPARP was demonstrated in three independent experiments. (**B**) Hepatocytes were incubated for 30–36 h with MT or WT Cholix in the presence of the indicated inhibitors, and cell viability was measured using the Cell Counting Kit 8. All data are presented as the mean ± standard deviation (SD). * *p* < 0.05, ** *p* < 0.005, *** *p* < 0.0001, ns: not significant.

To determine the role of EV-related pathways in Cholix-induced cell death, we first investigated the effect of general EV inhibitors (e.g., dimethyl amiloride [DMA], neticonazole hydrochloride [NH]). After incubating hepatocytes or HeLa cells with Cholix for 8 h in the presence or absence of EV inhibitors, Cholix-induced PARP cleavage was detected by Western blotting. Cholix-induced PARP cleavage in hepatocytes significantly increased in the presence of DMA and NH. Treatment with DMA or NH alone did not induce cell death (Figure 1A). However, these inhibitors did not affect Cholix-induced PARP cleavage in HeLa cells (Figure S1). We also investigated the effects of other EV inhibitors (GW4869, altenusin, desipramine, and imipramine) on Cholix-induced cell death. GW4869 and altenusin, which are neutral sphingomyelinase inhibitors [22,35], are inhibitors of EV generation [36]. Desipramine and imipramine are inhibitors of acid sphingomyelinase (SMPD1), which is involved in EV formation and release [33], norepinephrine reuptake, serotonin reuptake, and dopamine transport [37,38]. Hepatocytes were incubated with Cholix in the presence or absence of GW4869, and PARP cleavage was examined by Western blotting. Cholix-induced PARP cleavage was significantly suppressed by co-treatment with GW4869 but not with altenusin (Figure 1A). Notably, desipramine and imipramine significantly increased Cholix-induced PARP cleavage (Figure 1A). GW4869 inhibited Cholix-induced PARP cleavage in HeLa cells. Desipramine alone did not affect the Cholix-induced PARP cleavage (Figure S2).

We examined the effects of EV inhibitors on Cholix-induced cell death (Figure 1B). Consistent with the Western blotting results, cell viability significantly increased in the presence of GW4869. Incubation of hepatocytes with DMA, NH, desipramine, or imipramine along with Cholix decreased cell viability. We observed a significant change in cell shape after 24 h of Cholix treatment in the presence of desipramine. These findings suggest that the suppression of inhibitors (e.g., DMA, NH, imipramine, and desipramine) promotes Cholix-induced cell death. Thus, EV-related pathways may be involved in cell survival in the presence of Cholix.

To further investigate whether other sphingolipid metabolic pathways are involved in Cholix-induced cell death, we used several inhibitors (D609, ABC294640, FB1, and myriocin; Figure S3A) [39]. Inhibitors of ceramide synthase (FB1), serine palmitoyl transferase (myriocin), and sphingosine kinase-2 (ABC294640) did not affect the cleavage of Cholix-induced PARP, which was slightly suppressed by D609 (phosphatidyl choline-specific phospholipase C) (Figure S3B).

Taken together, these findings suggest that sphingolipid metabolic pathways are not involved in Cholix-induced cell death.

### 2.2. Effects of Z-VAD and Apaf-1 Knockdown on Desipramine-Enhanced Cholix-Induced Apoptosis

**Figure 2:**
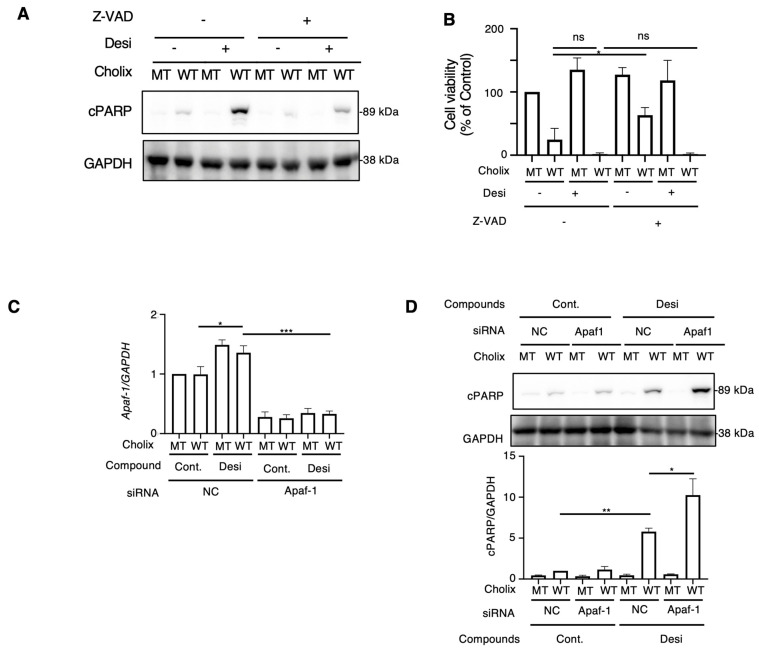
Effects of apoptosis inhibitor and Apaf1 knockdown on Cholix-induced cell death. (**A**) Hepatocytes (3 × 10^4^ cells/48-well plate) were incubated for 8 h with MT or WT Cholix with or without 10 μM desipramine (Desi) in the presence or absence of 10 μM Z-VAD-FMK. The cell lysates were subjected to Western blotting with the indicated antibodies. (**B**) Hepatocytes were incubated for 30-36 h with MT or WT Cholix in the presence of the indicated inhibitors, and cell viability was measured using the Cell Counting Kit 8. All data are presented as the mean ± standard deviation (SD). * *p* < 0.05, ** *p* < 0.005, *** *p* < 0.0001, ns: not significant. (**C**,**D**) Hepatocytes (3 × 10^4^ cells/48-well plate) were incubated for 8 h with MT or WT Cholix, with or without 10 μM desipramine (Desi) in control (NC) or *Apaf-1* siRNA-treated cells. Purified total RNA was subjected to a reverse transcription-quantitative polymerase chain reaction (RT-qPCR) using the indicated primers. β-actin (actin) was used as an internal control (**C**). The cell lysates were subjected to Western blotting using the indicated antibodies. Densitometric analysis of cPARP was performed for three independent experiments (**D**).

Next, to examine whether desipramine-enhanced Cholix-induced apoptosis is caspase-dependent or -independent, cells were incubated with Cholix in the presence or absence of the caspase inhibitors Z-VAD or desipramine. Cholix-induced PARP cleavage in control cells was significantly suppressed by Z-VAD, which significantly inhibited the desipramine-induced increase in Cholix-induced PARP cleavage (Figure 2A), suggesting that desipramine-enhanced apoptosis is regulated by a caspase-dependent pathway. We also investigated cell viability when cells were incubated for 30–36 h with Cholix in the presence or absence of the caspase inhibitors Z-VAD or desipramine. Consistent with the results shown in Figure 2A, Cholix-induced cell death was not inhibited even in the presence of Z-VAD. Z-VAD did not suppress desipramine- or Cholix-induced cell death, suggesting that early cell death could be inhibited by Z-VAD, but late cell death enhanced by desipramine was not sufficiently inhibited by Z-VAD (Figure 2B).

The caspase-dependent apoptotic pathway occurs via mitochondrial- or mitochondria-independent pathways [40]. Apoptotic peptidase-activating factor 1 (Apaf-1) is essential for intrinsic and mitochondria-dependent apoptosis [41]. To explore the cell death pathway activated by desipramine, we hypothesized that if desipramine acts as an activator of the intrinsic apoptotic pathway, Apaf-1 knockdown would downregulate the Cholix-induced apoptotic pathway enhanced by desipramine. Reverse transcription-quantitative polymerase chain reaction (RT-qPCR) confirmed that Cholix slightly increased *Apaf-1* mRNA levels, which decreased in siRNA-transfected cells (Figure 2C). We observed that the increase in Cholix-induced PARP cleavage after desipramine treatment was not inhibited but rather promoted in Apaf-1-knockdown cells, indicating that the desipramine-stimulated apoptotic pathway may be mitochondria-independent (Figure 2D).

### 2.3. Effects of Extracellular Vesicle-Regulating Protein Knockdown on Cholix-Induced Cell Death

**Figure 3:**
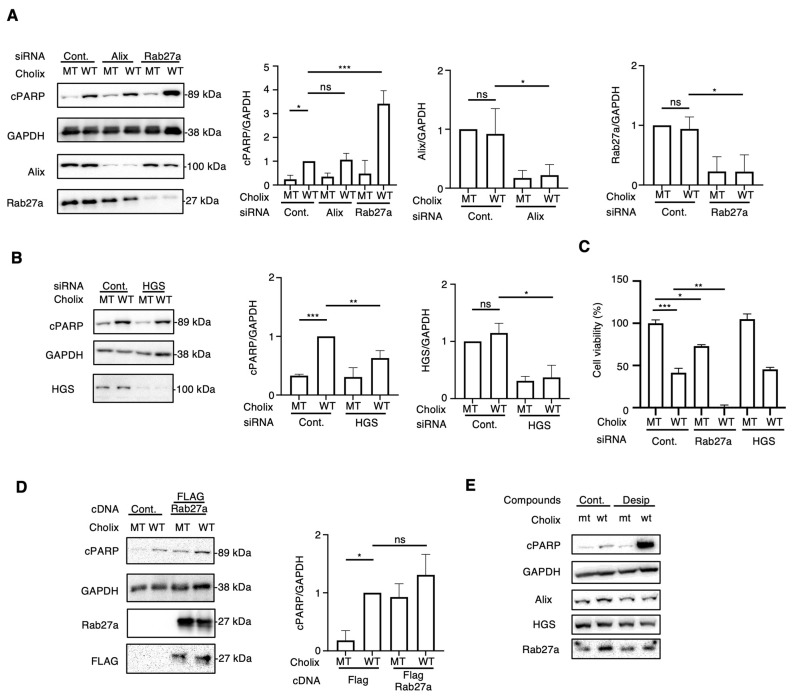
Effects of knockdown of extracellular vesicle-regulating proteins on Cholix-induced cell death. (**A**,**B**) Hepatocytes transfected with the indicated siRNAs (3 × 10^4^ cells in 48-well plates) were incubated for 8 h with MT or WT Cholix. Cell lysates were subjected to Western blotting using anti-cPARP, anti-Alix, or anti-Rab27a antibodies. GAPDH was used as an internal control. Densitometric analysis of cPARP was performed for three independent experiments. (**C**) The indicated siRNA-transfected cells (1 × 10^4^ cells/96-well plate) were incubated for 30–36 h with MT or WT Cholix in the presence of the indicated inhibitors, and cell viability was measured using the Cell Counting Kit 8. (**D**). Control- or FLAG-tagged Rab27a-transfected hepatocytes (3 × 10^4^ cells/48-well plate) were incubated for 8 h with MT or WT Cholix. The cell lysates were subjected to Western blotting using the indicated antibodies. Densitometric analysis of cPARP was performed for three independent experiments. All data are presented as the mean ± SD. * *p* < 0.05, ** *p* < 0.005, *** *p* < 0.0001, ns: not significant. (**E**). Hepatocytes (3 × 10^4^ cells/48-well plate) were incubated for 8 h with MT or WT Cholix, with or without 10 μM desipramine (Desi). The cell lysates were subjected to Western blotting using the indicated antibodies.

Incubation of hepatocytes with EV inhibitors (DMA, NH, desipramine, and imipramine) enhanced Cholix-induced cell death. Next, we investigated the EV-regulating proteins responsible for Cholix-induced cell death. Previous studies have identified crucial proteins (e.g., Rab27a [19], Alix [20], SMPD1, SMPD2 and 3 [21,22], and HGS [23]) involved in EV biogenesis and trafficking. To investigate whether EV-associated molecules participate in Cholix-induced cell death, we depleted Rab27a, Alix, SMPD1, SMPD2, SMPD3, and HGS in hepatocytes using small interfering RNAs (siRNAs) and examined their effects on Cholix-induced cell death. We detected significantly decreased mRNA levels of *Rab27a*, *Alix*, *SMPD2*, and *HGS*, but not *SMPD1* or *3* in the siRNA-transfected cells (Figure 3A,B and Figure S4A). SMPD1 and 3 mRNAs were barely detectable in hepatocytes using RT-qPCR. Notably, SMPD1, a target of desipramine and imipramine, was not expressed in hepatocytes, suggesting that a novel effect of desipramine promotes the Cholix-induced cell death pathway.

We next observed that Cholix-induced PARP cleavage was significantly increased in Rab27a-knockdown cells but not in Alix-knockdown cells (Figure 3A). SMPD2 knockdown did not affect Cholix-induced PARP cleavage, which is inconsistent with the results obtained in GW4869-treated cells (Figure S4B). HGS knockdown slightly inhibited Cholix-induced PARP cleavage (Figure 3B) compared with the non-targeting control siRNA. Next, we assessed the viability of siRNA-transfected cells transfected with mutant (MT) or wild-type (WT) Cholix (Figure 3C). Cell viability after HGS knockdown exhibited a pattern similar to that observed in control siRNA-transfected cells. In the presence of Cholix, a significant increase in cell death upon Rab27a knockdown was observed compared with control cells.

Next, we examined the effect of Cholix on FLAG-tagged Rab27a-overexpressing hepatocytes. Cholix-induced PARP cleavage was slightly, but not significantly, increased in Rab27a-overexpressing cells compared with that in control cells (Figure 3D). We investigated whether desipramine affects the expression of Rab27a in hepatocytes (Figure 3E). We found that Rab27a expression was not altered in the presence of Cholix or desipramine.

These data suggest that Rab27a participates in the Cholix-induced caspase-dependent cell death pathway and that its endogenous expression is sufficient for the activation of cell death signaling. Desipramine-activated Cholix-induced apoptotic signaling is controlled by a Rab27a-independent pathway.

### 2.4. RNA Sequencing Analysis of the Desipramine-Promoted Cholix-Induced Cell Death Pathway

**Figure 4:**
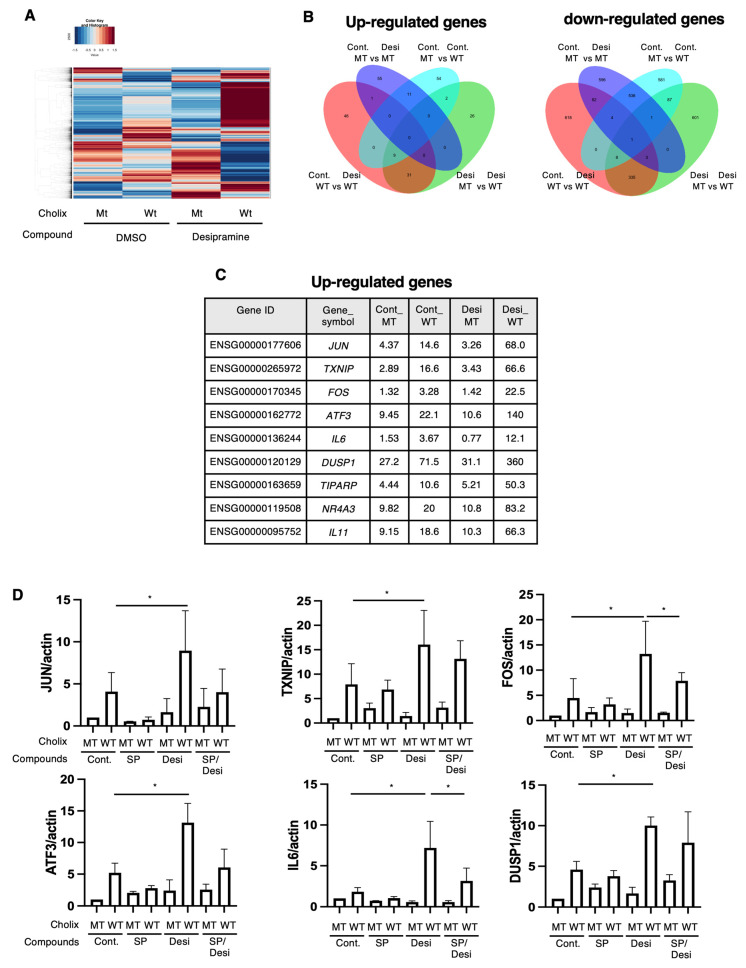
RNA sequencing analysis of Cholix-induced cell damage in the presence of desipramine. (**A**) The hierarchical clustering of differentially expressed genes (DEGs) in the four comparisons identified using RNA sequencing. Hepatocytes were incubated with MT or WT Cholix in the presence or absence of desipramine. (**B**) Venn diagram displaying the overlap of DEGs (left, upregulated; right, downregulated). (**C**) The specific upregulated genes are listed in the table. (**D**) Hepatocytes (1 × 10^5^ cells in a 12-well plate) were incubated for 7–8 h with MT(Cm) or WT Cholix (Cw), with or without 10 μM JNK inhibitor, SP600125 (SP), in the presence or absence of 10 μM desipramine (Desi). Purified total RNAs were subjected to RT-qPCR using the indicated primers. β-Actin (actin) was used as an internal control. The cell lysates were subjected to Western blotting using the indicated antibodies. GAPDH was employed as an internal control. Densitometric analysis of cPARP was performed for three independent experiments. Data are presented as the mean ± SD. * *p* < 0.05.

To clarify the function of desipramine in the Cholix-induced cell death pathway, we identified desipramine-enhanced cell death pathway-related genes using RNA sequencing (RNA-seq). We retrieved the data and generated a heatmap (Figure 4A) and a Venn diagram of the upregulated (Figure 4B, left panel) and downregulated (Figure 4B, right panel) genes. We identified nine commonly upregulated genes in the Cholix- and Cholix/desipramine-treated cells (Figure 4C). Cholix-upregulated genes (e.g., *JUN*, *TXNIP*, *FOS*, *ATF3*, *IL6*, *DUSP1*, *TIPARP*, *NR4A3*, and *IL11*) showed enhanced expression in the presence of desipramine. Cholix-downregulated genes (e.g., *MYCL* and *PPP1R3G*) also showed reduced expression levels following desipramine treatment.

Ogura et al. demonstrated that Cholix-induced apoptosis involves a c-Jun N-terminal protein kinase (JNK)-dependent pathway [13]. To investigate whether desipramine enhanced Cholix-induced JNK activation, we confirmed the expression levels of the upregulated genes using RT-qPCR in the presence of the JNK inhibitor SP600125. In agreement with the RNA-seq data, the addition of desipramine enhanced the expression levels of *JUN*, *TXNIP*, *FOS*, *ATF3*, *IL6*, *DUSP1*, *TIPARP*, *NR4A3*, and *IL11* mRNA, which were downregulated in the presence of the JNK inhibitor SP600125 (Figure 4D). These data suggest that desipramine enhances Cholix-mediated JNK activation.

### 2.5. Analysis of the Desipramine-Enhanced Cholix-Induced Cell Death Pathway

**Figure 5:**
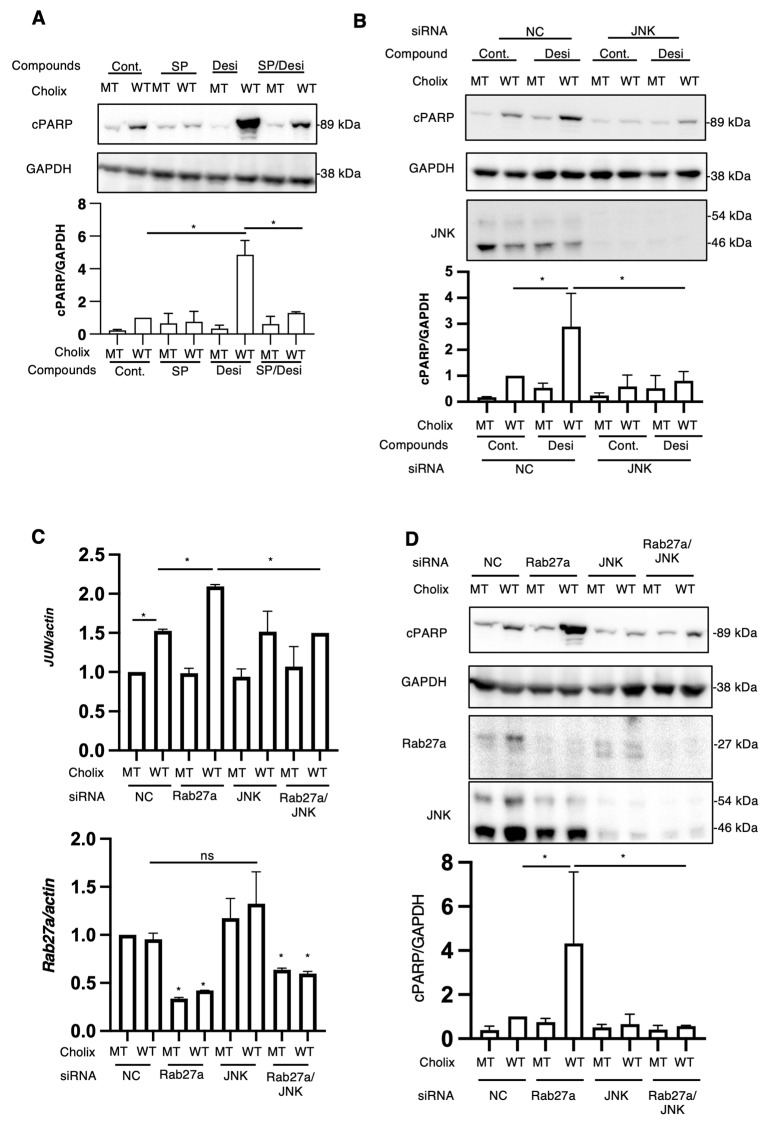
Effects of JNK inhibitors or JNK knockdown on Cholix-induced apoptotic pathway. (**A**) Hepatocytes (3 × 10^4^ cells/48-well plate) were incubated for 7–8 h with MT or WT Cholix in the presence or absence of SP601125 (SP) and with or without desipramine (Desi). The cell lysates were subjected to Western blotting using anti-cPARP antibodies. GAPDH was used as an internal control. Densitometric analysis of cPARP was performed for three independent experiments. Data are presented as the mean ± SD. * *p* < 0.05. (**B**) Hepatocytes transfected with JNK siRNA (3 × 10^4^ cells/48-well plate) were incubated for 8 h with MT or WT Cholix in the presence or absence of 10 μM desipramine (Desi). Cell lysates were subjected to Western blotting using antibodies against cPARP and JNK. GAPDH was employed as an internal control. Densitometric analysis of cPARP was performed in three independent experiments, as described below. Data are presented as the mean ± SD. * *p* < 0.05. (**C**,**D**) Hepatocytes transfected with the indicated siRNAs (1 × 10^5^ cells/12-well plate) were incubated for 7–8 h with Cholix. Purified total RNAs were subjected to RT-qPCR using the Jun and Rab27a primers. β-Actin (actin) was employed as an internal control (**C**). Cell lysates were subjected to Western blotting with antibodies against cPARP, Rab27a, and JNK. GAPDH was employed as an internal control. Densitometric analysis of cPARP was performed in three independent experiments (**D**). Data are presented as the mean ± SD. * *p* < 0.05. All data are presented as the mean ± SD. * *p* < 0.05, ns: not significant.

The RNA-seq results indicated an increase in the expression levels of JNK-associated genes, as described earlier. We investigated the effects of SP600125 on Cholix-induced PARP cleavage in the presence of desipramine. As shown in Figure 5A, desipramine-enhanced Cholix-induced PARP cleavage was significantly suppressed in the presence of SP600125.

We hypothesized that desipramine inhibition or Rab27a knockdown would stimulate JNK, followed by the promotion of Cholix-induced PARP cleavage. To investigate whether the JNK-dependent pathway participates in the cytotoxicity of Cholix, cells treated with the JNK inhibitor SP600125 or JNK-siRNA-transfected cells were incubated with Cholix in the presence or absence of desipramine (Figure 5A,B). Desipramine-enhanced PARP cleavage by Cholix was significantly decreased in SP600125-treated and JNK-knockdown cells.

To understand why Cholix-induced PARP cleavage was enhanced in Rab27a-knockdown cells, we hypothesized that the JNK pathway was activated by Rab27a knockdown. Therefore, we investigated the effect of JNK on the Cholix-stimulated apoptotic pathway in Rab27a-knockdown cells. After hepatocytes were transfected with *Rab27a* siRNA, with or without JNK siRNA, the cells were treated with Cholix. The enhanced PARP cleavage by Cholix in Rab27a-knockdown cells was significantly decreased in Rab27a and JNK double-knockdown cells (Figure 5C). To investigate whether Rab27a knockdown involves a Cholix-mediated increase in *Jun* mRNA levels, we transfected *Rab27a* siRNA with or without JNK siRNA and added Cholix. The results suggested that Rab27a knockdown significantly decreased *Rab27a* mRNA levels and enhanced Cholix-induced *Jun* mRNA expression, which was inhibited by JNK siRNA (Figure 5D). These findings suggest that the inhibition of Rab27a-dependent signaling and desipramine has a priming effect on the JNK pathway, which enhances Cholix-induced apoptosis. 

IL11 mRNA was increased in Cholix-treated hepatocytes treated with desipramine. Widjaja et al. reported that species-matched IL11 secreted from acetaminophen-damaged human and mouse hepatocytes generates an autocrine loop of NADPH-oxidase-4-dependent cell death [42]. We investigated the secondary effect of IL11 on cell death induced by Cholix. Recombinant human IL11 was added to hepatocytes, which were then incubated with or without Cholix in the presence or absence of desipramine (Figure S5). We observed that the addition of IL11 did not affect Cholix-induced PARP cleavage.

## 3. Discussion

EV inhibitors affect different stages of the EV biogenesis signaling pathway [34]. Imipramine and desipramine, which enhanced Cholix-induced cell death (Figure 1A), suppresses the biogenesis pathway of early to late EVs by blocking acid sphingomyelinase activity [43], although SMPD1 was not detected in hepatocytes. These data indicated that the novel function of these drugs was to enhance Cholix-induced cell death.

In our study, we found that co-treatment with desipramine and Cholix significantly increased JNK-related transcription factor mRNA levels compared with treatment with Cholix alone (Figure 4C). The levels of these upregulated genes and PARP cleavage were dramatically reduced in the presence of the JNK inhibitor SP600125 (Figure 5A) and in JNK-knockdown cells (Figure 5B), although desipramine alone did not increase JNK-regulated mRNA.

JNK is an essential mediator of the apoptotic pathway. JNK plays an important role in both canonical and non-canonical apoptotic signaling pathways [44,45]. In the mitochondria-dependent canonical apoptotic pathway, Apaf-1 forms a complex with cytochrome c in the cytosol and activates caspase-dependent apoptosis [46]. Tran et al. reported that oxidant-activated JNK interacts with Apaf1 and cytochrome c, which delays the formation of active apoptosome, thereby protecting cells [47]. Another study showed a direct interaction between JNK and the anti-apoptotic protein apurinic/apyrimidinic endonuclease 1 (APE1) in cells undergoing apoptosis, which is associated with the E3 ligase ITCH, followed by the ubiquitination of APE1 and degradation, resulting in cell death [48]. Notably, Apaf-1 knockdown promoted JNK-mediated Jun and Fos mRNA expression in the presence of Cholix and enhanced Cholix-induced PARP cleavage in the presence of desipramine (Figure S6). Imao et al. reported that staurosporine-induced apoptosis in lymphoid and myeloid cells was regulated by non-canonical Apaf-1- and caspase8-independent signaling pathway, which were suppressed in the presence of a serine/threonine phosphatase inhibitor [49]. Lassus et al. showed that Apaf-1, caspase-2, and caspase-9 are not essential for programmed cell death in factor-dependent cells [50]. These findings suggest that the interaction between JNK and Apaf1 may have an inhibitory effect not only on the Apaf1-dependent canonical apoptosis pathway but also on the JNK-dependent Apaf1-independent noncanonical apoptosis pathway. In the presence of desipramine, Cholix-induced apoptosis was mediated through the JNK-dependent non-canonical apoptosis pathway.

Neticonazole hydrochloride (NH), an antifungal and antitumor compound, blocks the EV formation signaling pathway by suppressing Alix, Rab27a, and nSMase2 [51]. In hepatocytes, Cholix-induced cell death was enhanced by knockdown of Rab27a but not by Alix, SMPD2, or HGS (Figure 2A,B). Our findings suggested that the Rab27a-dependent pathway plays a crucial role in Cholix-induced cell death. Cholix-induced *c-Jun* mRNA levels significantly increased in Rab27a-knockdown cells and decreased in cells co-transfected with JNK siRNA. These results indicated that the decreased level of Rab27a induced a priming effect on JNK, which was activated in the presence of Cholix, resulting in the promotion of the Cholix-induced apoptosis pathway.

DMA, an inhibitor of the EV formation pathway that impairs calcium (H^+^/Na^+^ and Na^+^/Ca^2+^) channels [52], also enhances the Cholix-induced apoptosis signaling pathway. Previous studies reported that DMA prevents thapsigargin-induced apoptosis via intracellular alkalinization [53] and attenuates lethal reperfusion injury in the ventricular myocardium during reperfusion [54]. In contrast to the effects of DMA observed in the presence of Cholix, DMA is a functional agent that promotes cell death. The potential synergistic effect of the stimulation resulting from the inhibition of protein synthesis by Cholix and the inhibition of channel formation by DMA on cell death requires further elucidation to better understand the underlying mechanisms, including the JNK signaling pathway.

GW4869 suppressed the biogenesis of late EVs in a manner similar to that of imipramine or desipramine. This inhibitor partially suppressed the Cholix-induced apoptotic pathway (PARP cleavage) and cell death (Figure 1A,B). Inhibition of N-SMase activation by GW4869 suppressed the TNFα-mediated canonical apoptosis pathway [35]. Knockdown of sphingomyelinases (SMPD 1 or 2/3) by siRNA did not affect Cholix-induced PARP cleavage (Figure S4A,B), suggesting that sphingomyelinases are not involved in the Cholix-induced cell death pathway. However, the signaling pathways directly inhibited by GW4869 remain unknown. The inhibitory effect of GW4869 on Cholix-induced cell death may involve EV secretion rather than cell signaling pathways. In this study, we clarified this point using EVs purified from cells in the next project.

The sphingomyelin synthase inhibitor, D609, partially suppressed Cholix-induced PARP cleavage (Figure S3B) and partially inhibited Cholix-induced JNK-regulated mRNA (*Jun*, *IL-6*, *FOS*) expression when combined with desipramine (Figure S3C). D609 is also known as an antioxidant or phosphatidylcholine-specific phospholipase inhibitor [55]. Cholix increases reactive oxygen species (ROS) generation in hepatocytes [12]. ROS are known stimulators of JNK [56]. These findings suggest that the effect of D609 is suppressed by Cholix-increased ROS, followed by partial inhibition of the JNK signaling pathway, resulting in a slight inhibition of the desipramine-enhanced Cholix-induced apoptotic signaling pathway.

Z-VAD-FMK is a cell-permeable and irreversible pan-caspase inhibitor that blocks caspase-dependent apoptosis in several cell lines. Cholix-induced PARP cleavage was inhibited by Z-VAD [57] and the enhancement in PARP cleavage by desipramine (Figure 2A). In contrast, cell viability analysis showed that Cholix-induced cell death was partially suppressed by Z-VAD; however, the desipramine-mediated enhancement in Cholix-induced cell death was not inhibited by Z-VAD (Figure 2B). These results suggest that Cholix-induced cell death involves not only canonical apoptosis mediated by caspase-dependent cleavage of PARP but also other cell death mechanisms that are enhanced by desipramine. In addition to apoptosis, various programmed cell death mechanisms, including pyroptosis, ferroptosis, necroptosis, and autophagic cell death, have received increasing attention, and accumulating experimental evidence has revealed that cell death is cross-regulated by various stimuli and environmental conditions [58]. Our previous study showed that the necroptosis inhibitor Necrostatin-1 did not suppress Cholix/TNF-α-induced cell death13, suggesting that necroptosis is not involved in the enhancement of cell death by desipramine. As proposed in our study, analysis of the enhancement in Cholix-induced cell death by the inhibition of EV formation signals may reveal a novel crosstalk in cell death signaling.

## 4. Materials and Methods

### 4.1. Preparation of Purified Cholix Toxin

WT Cholix and catalytically inactivated MT Cholix (E581) were purified as described previously [11].

### 4.2. Antibodies, Reagents, Plasmids, and siRNAs

Antibodies against cleaved PARP (Asp214) (#5625) and Rab27a (#69295) were purchased from Cell Signaling Technology (Danvers, MA, USA). The anti-GAPDH antibody (#10494-1-AP) was purchased from ProteinTech (Rosemont, IL, USA), and the anti-Alix antibody (#634502) was purchased from BioLegend (San Diego, CA, USA). Anti-HGS antibody (#GTX101718) was obtained from GeneTex (Irvine, CA, USA). Horseradish peroxidase (HRP)-conjugated goat anti-rabbit (#HAF008) and anti-mouse (#HAF007) secondary antibodies were purchased from R&D Systems (Minneapolis, MN, USA).

GW4869 (#13127), altenusin (#26911), DMA (#19100), imipramine (#15890), and SP600125 (#10010466) were purchased from the Cayman Chemical Company (Ann Arbor, MI, USA). The general caspase inhibitor Z-VAD-FMK (#550377) was obtained from the Peptide Institute. Desipramine hydrochloride (#042-33931) was obtained from FUJIFILM Wako Chemicals (Tokyo, Japan), and neticonazole hydrochloride (#S4878) was obtained from Selleck Chemicals (Houston, TX, USA). Universal negative control siRNA was purchased from Nippon Gene (Tokyo, Japan). Other siRNAs were purchased from Qiagen (Hilden, Germany) or Thermo Fisher Scientific (Waltham, MA, USA) or synthesized by Sigma-Aldrich (St Louis, MO, USA) (Figure S1).

FLAG-tagged or GFP-tagged Rab27a expression plasmids were constructed by amplifying the Rab27a sequence from cDNA isolated from HeLa cells, using the primers listed in Table S1. The PCR products were inserted into the NotI and SalI multicloning sites of the FLAG-5a vector (Sigma-Aldrich) using an In-Fusion HD cloning kit (Takara Bio Inc., Shiga, Japan).

### 4.3. Cell Culture and Transfection

Immortalized human hepatocytes [59], kindly provided by Dr. Tatsuo Kanda (Nihon University School of Medicine, Tokyo, Japan), were cultured in RPMI-1640 medium (Sigma-Aldrich) supplemented with 10% heat-inactivated fetal bovine serum and penicillin/streptomycin (Sigma-Aldrich).

Cells were plated overnight and transfected for 48 h with 50–100 nM control or the indicated siRNAs using Lipofectamine RNAiMax reagent (Thermo Fisher Scientific), or overexpression plasmids using Polyethylenimine Max (Polysciences, Warrington, MA, USA) in RPMI640 without serum, as previously reported [12]. The sequences of the target siRNAs are listed in Table S1.

### 4.4. Total RNA Purification, RNA-Seq, and RT-qPCR Analyses

Hepatocytes were incubated for 7–8 h with Cholix in the presence or absence of the indicated compounds, and RNA was purified from human hepatocytes using ISOGEN II or ISOGEN (NIPPON GENE, Tokyo, Japan) according to the manufacturer’s instructions. cDNA was synthesized using PrimeScript™ RT Master Mix (TaKaRa Bio, Shiga, Japan). Paired-end RNA-seq libraries were constructed using the NEBNext Poly(A) mRNA Magnetic Isolation Module (#E7490) and the NEBNext UltraTM II Directional RNA Library Prep Kit (#E7760) and then sequenced using a NanoSeq 6000 platform (Illumina, San Diego, CA, USA). Sequencing was performed using Rhelixa (Tokyo, Japan), and paired-end reads of 150 bp × 2 were obtained.

To evaluate the mRNA expression levels, qPCR analysis was performed using the KOD SYBER qPCR Mix (TOYOBO, Tokyo, Japan) and the Thermal Cycle Dice Real-Time System III (Takara Bio). The primers used for the PCR are listed in Table S1.

### 4.5. Immunoblotting Analysis

Hepatocytes were incubated for 7–8 h with 1–5 μg mL^−1^ Cholix in the presence or absence of the indicated compounds, and then the cells were lysed with 1× sodium dodecyl sulfate (SDS) sample buffer and then heated for 10 min at 100 °C. After SDS-polyacrylamide gel electrophoresis (PAGE), proteins were transferred to polyvinylidene fluoride (PVDF) membranes (#IPVH00010; Merck, Darmstadt, Germany), blocked with 5% skim milk (#190-12865; WAKO) in Tris-buffered saline with Tween 20 (TTBS), and then incubated with antibodies diluted in Immuno Shot Reagent (Cosmo Bio, Tokyo, Japan) overnight at 4 °C. The membranes were washed three times with TTBS and incubated for 1 h with horseradish peroxidase-conjugated secondary antibodies (R&D Systems), followed by enhanced chemiluminescence detection using EzWestLumi One (ATTO, Tokyo, Japan). Bands were detected and densitometric analysis was performed using a WES-6100 LuminoGraph I instrument (ATTO).

### 4.6. Cell Viability Assay

Hepatocytes were cultured in 96-well plates overnight and then incubated for 30 h with the indicated inhibitors in the presence of 5 μg mL^−1^ MT or WT Cholix in 100 μL of serum-free RPMI1640. After washing the plate thrice with PBS, the reaction buffer of the Cell Counting Kit-8 assay (Dojindo, Kumamoto, Japan) was added to each well. The samples were incubated for 1 h at 37 °C, and the absorbance was measured at 450 nm using a Varioskan plate reader (Thermo Fisher Scientific).

### 4.7. Statistics and Reproducibility

All experiments were performed at least in triplicates, and similar results were obtained for each replicate. The RT-qPCR assays and densitometric data were analyzed using Student’s t-test or one-way ANOVA, followed by Dunnett’s test using GraphPad Prism 9 software (GraphPad, San Diego, CA, USA). Statistical significance was set at *p*-value < 0.05.

## Data Availability

Raw sequencing read data were deposited in the DNA Data Bank of Japan (BioProject accession number: PRJDB17473). The BioSample and Run IDs are listed in Table S2.

## Supplementary Materials

The following supporting information can be downloaded at https://www.mdpi.com/article/10.3390/toxins16090380/s1, Figure S1: Effects of general extracellular vesicle inhibitors on Cholix-induced HeLa cell death; Figure S2: Effects of sphingomyelinase inhibitors on Cholix-induced HeLa cell death; Figure S3: Effects of sphingolipid metabolic pathway inhibitors on Cholix-induced cell death; Figure S4: Verification of the inhibitory effects of the siRNAs; Figure S5: Effects of IL11 on Cholix-induced cell death pathway; Figure S6: Effects of JNK inhibitor and Apaf1 knockdown on desipramine-enhanced Cholix-activated JNK-regulating mRNA. Table S1: Sequences of primers for RT-qPCR, construct for expression plasmid and siRNA oligonucleotides; Table S2: Raw read data obtained in this study. Refs. [60,61,62,63] cited in Supplementary.
