# Supplementary material for: Extracellular Vesicle Inhibitors Enhance Cholix-Induced Cell Death via Regulation of the JNK-Dependent Pathway
Source: Toxins (Basel). 2024 Aug 29;16(9):380. doi: 10.3390/toxins16090380 (PMC11435833; doi:10.3390/toxins16090380)
Supplement: Supplementary file 1 [file toxins-16-00380-s001.zip › Supplymental Figure legends.pdf]

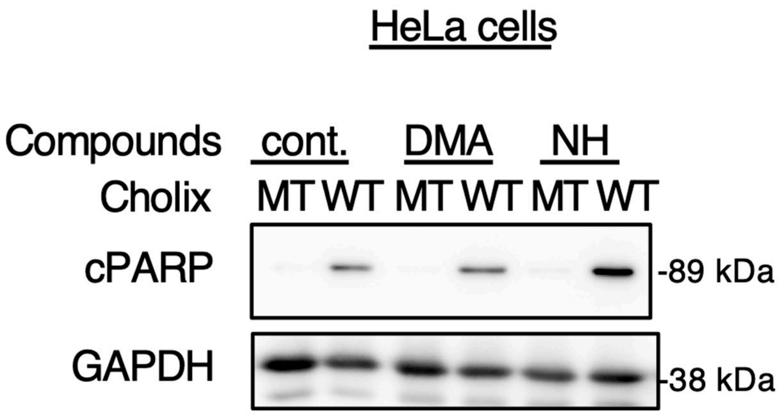

Figure S1. Effects of general extracellular vesicle inhibitors on Cholix-induced HeLa cell death. HeLa cells (3×10^4 cells/48-well plate) were incubated for 8 h with catalytically inactivated mutant (MT) or wild-type (WT) Cholix in the presence or absence of 0.1% dimethyl sulfoxide (DMSO; control), 10 μM dimethyl amiloride (DMA), or 10 μM neticonazole hydrochloride (NH). Proteins from the cells were subjected to Western blotting using an anti-cleaved PARP (cPARP) antibody. GAPDH was used as an internal control. Densitometric analysis of cPARP was performed for three independent experiments. Data are presented as mean ± standard deviation (SD). $^*P < 0.05$, ns: not significant.

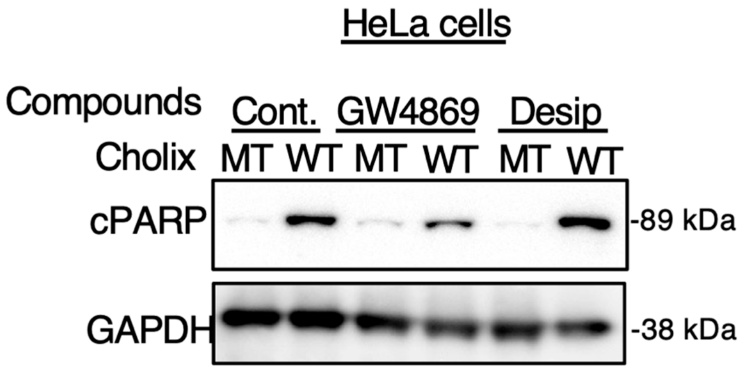

Figure S2. Effects of sphingomyelinase inhibitors on Cholix-induced HeLa cell death. HeLa cells (3 × 10^4 cells/48-well plate) were incubated for 8 h with MT or WT Cholix in the presence or absence of DMSO, 10 µM GW4869, or 10 µM desipramine. Cell proteins were subjected to Western blotting using an anti-cPARP antibody. GAPDH was used as an internal control. Densitometric analysis of cPARP was performed for three independent experiments. Data are presented as the mean ± SD. *$P < 0.05$, ns: not significant.

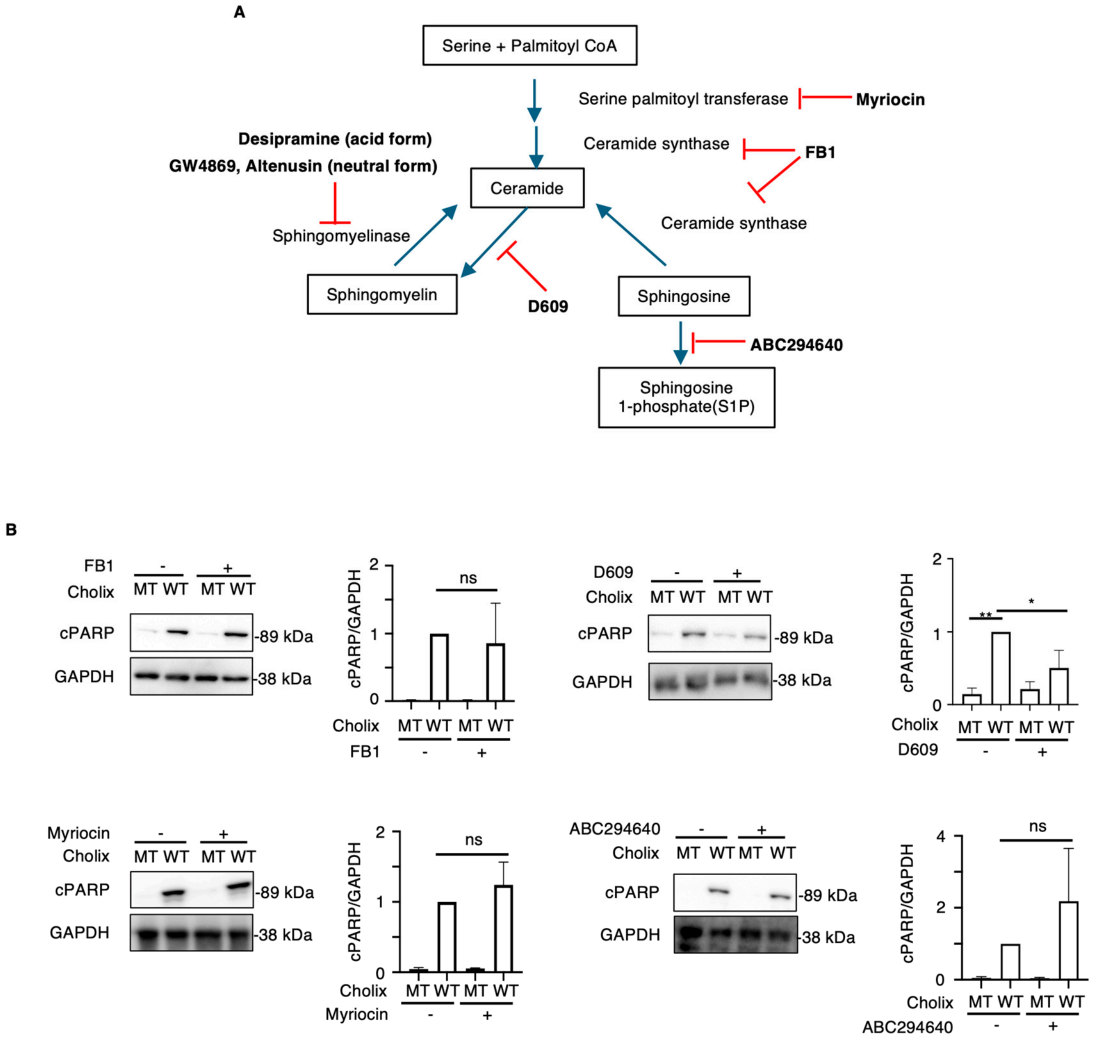

Figure S3. Effects of sphingolipid metabolic pathway inhibitors on Cholix-induced cell death. **A.** The pathways of ceramide synthesis and its inhibitors. Inhibitors of sphingomyelin synthesis, sphingosine kinases, and their pathways were modified as previously described [60]. **B.** Hepatocytes (3 × 10^4 cells/48-well plate) were incubated for 8 h with MT or WT Cholix in the presence or absence of DMSO (control), 10 μM FB1, 10 μM D609, 10 μM myriocin, or 10 μM ABC294640. Cell lysates were analyzed by Western blot using an antibody against cPARP. GAPDH was used as an internal control. Densitometric analysis of cPARP was performed for three independent experiments. Data are presented as the mean ± SD. *$P < 0.05$, ns: not significant. **C.** Hepatocytes (1 × 10^5 cells in a 12-well plate) were incubated for 7-8 h with MT(Cm) or WT Cholix (Cw), with or without 10 μM D609 in the presence or absence of 10 μM desipramine (Desi). The purified total RNAs were subjected to RT-qPCR using the indicated primers. β-Actin (actin) was used as an internal control. Data are presented as the mean ± SD. *$P < 0.05$, ns: not significant.

Reference

60. Cao, M.; Ji, C.; Zhou, Y.; Huang, W.; Ni, W.; Tong, X.; Wei, J.F. Sphingosine kinase inhibitors: A patent review. *Int. J. Mol. Med.* **2018**, *41*, 2450–2460. https://doi.org/10.3892/ijmm.2018.3505.

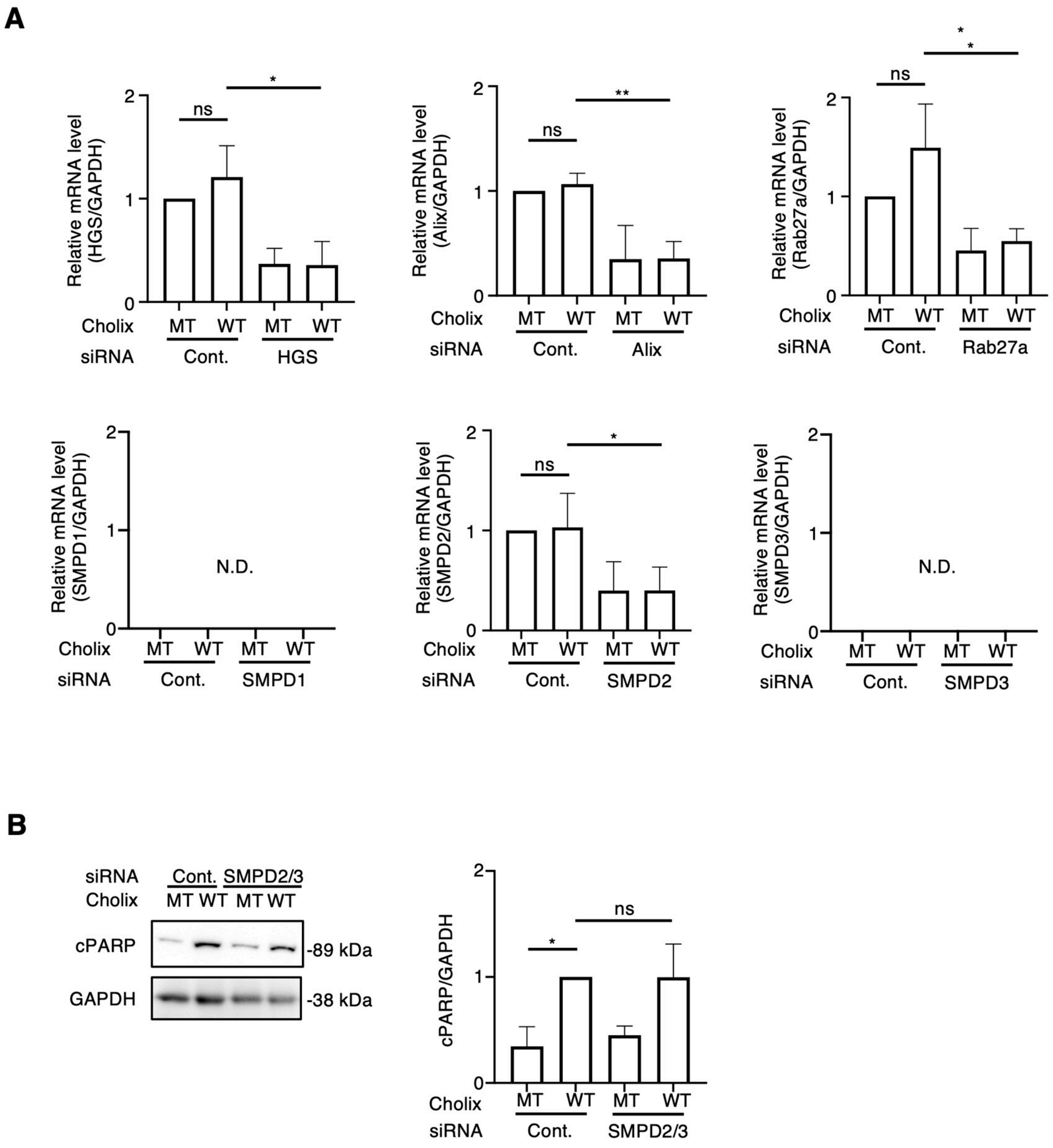

Figure S4. Verification of the inhibitory effects of the siRNAs. A. Effect of the indicated siRNAs on hepatocytes. Hepatocytes (1 × 10^5 cells/12-well plate) were transfected with the indicated siRNAs and incubated for 8 h with MT or WT Cholix. The mRNA expression levels were measured using reverse transcription-quantitative polymerase chain reaction. Data are presented as the mean ± SD. *P < 0.05, ***P < 0.001, ns: not significant, N.D: not detected. B. Effect of SMPD2/3 siRNA transfection on Cholix-induced PARP cleavage. Hepatocytes (3 × 10^4 cells/48-well plate) were transfected with the indicated siRNAs and incubated for 8 h with MT or WT Cholix. Cell proteins were subjected to Western blotting using an anti-cPARP antibody. GAPDH was used as an internal control. Densitometric analysis of cPARP was performed for three independent experiments. Data are presented as the mean ± SD. ***P < 0.001, ns: not significant.

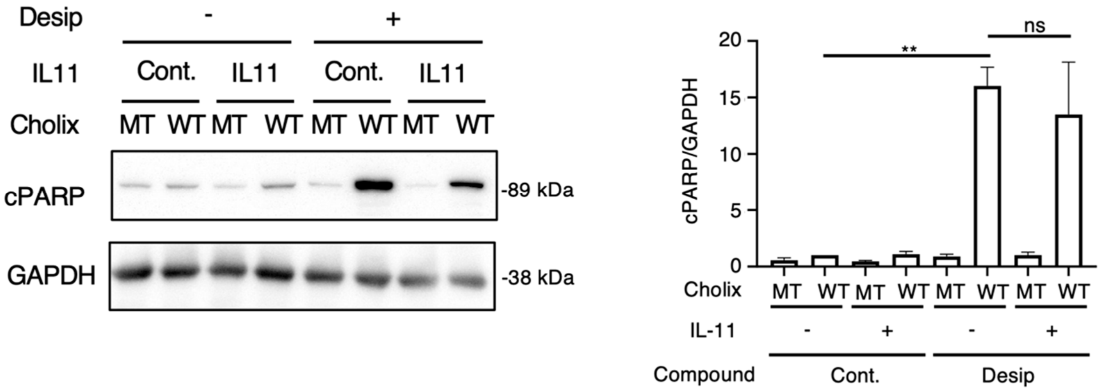

Figure S5. Effects of IL11 on Cholix-induced cell death pathway. Hepatocytes (3 × 10^4 cells/48-well plate) were incubated for 7-8 h with MT, WT Cholix, and/or IL11 in the presence or absence of desipramine (Desi). Cell lysates were subjected to Western blotting using anti-cPARP or anti-Apaf-1 antibodies. GAPDH was used as the internal control (left panel). Densitometric analysis of cPARP was performed for three independent experiments (right panel). Data are presented as the mean ± SD. $^{*}P < 0.05$.

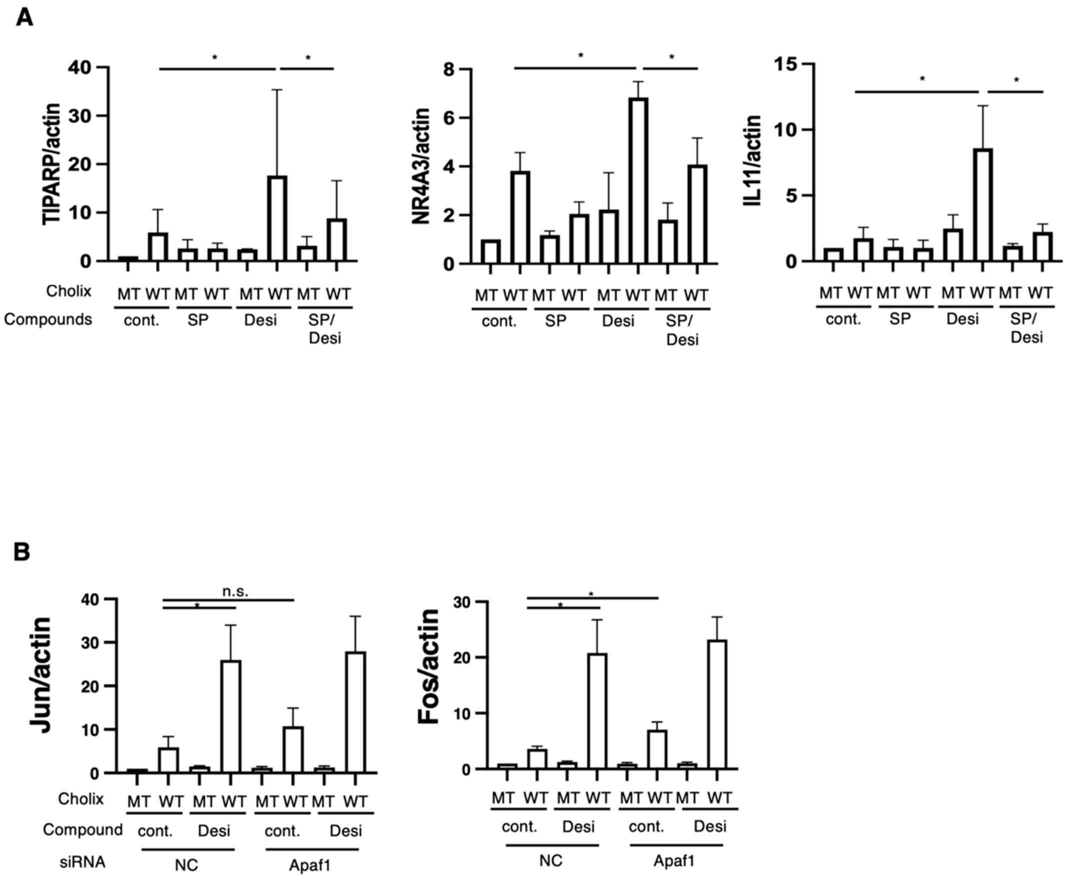

Figure S6. Effects of JNK inhibitor and Apaf1 knockdown on desipramine-enhanced Cholix-activated JNK-regulating mRNA. Hepatocytes (1 × 10^5 cells in a 12-well plate) were incubated for 7-8 h with MT(Cm) or WT Cholix (Cw), with or without the 10 μM JNK inhibitor SP600125 (SP) and in the presence or absence of 10 μM desipramine (Desi). The purified total RNAs were subjected to RT-qPCR using the indicated primers. β-Actin (actin) was used as an internal control. The indicated siRNA-transfected hepatocytes were incubated with 10 μM desipramine (Desi) in the presence of Cholix MT or WT for 8 h and then measured the expression level of Jun and Fos mRNA by qRT-PCR. β-Actin (actin) was used as an internal control. Data are presented as the mean ± SD. *$P < 0.05$, ns: not significant.
